# Electrocatalytic Hydrogenation of N_2_ to NH_3_ by MnO: Experimental and Theoretical Investigations

**DOI:** 10.1002/advs.201801182

**Published:** 2018-11-09

**Authors:** Zao Wang, Feng Gong, Ling Zhang, Rui Wang, Lei Ji, Qian Liu, Yonglan Luo, Haoran Guo, Yuehui Li, Peng Gao, Xifeng Shi, Baihai Li, Bo Tang, Xuping Sun

**Affiliations:** ^1^ Institute of Fundamental and Frontier Sciences University of Electronic Science and Technology of China Chengdu 610054 Sichuan China; ^2^ College of Chemistry Sichuan University Chengdu 610064 Sichuan China; ^3^ School of Materials and Energy University of Electronic Science and Technology of China Chengdu 611731 Sichuan China; ^4^ International Center for Quantum Materials and Electron Microscopy Laboratory School of Physics Peking University Beijing 100871 China; ^5^ Collaborative Innovation Centre of Quantum Matter Beijing 100871 China; ^6^ College of Chemistry Chemical Engineering and Materials Science Shandong Normal University Jinan 250014 Shandong China

**Keywords:** ambient conditions, artificial N_2_ fixation, electrocatalysis, MnO, NH_3_

## Abstract

NH_3_ is a valuable chemical with a wide range of applications, but the conventional Haber–Bosch process for industrial‐scale NH_3_ production is highly energy‐intensive with serious greenhouse gas emission. Electrochemical reduction offers an environmentally benign and sustainable route to convert N_2_ to NH_3_ at ambient conditions, but its efficiency depends greatly on identifying earth‐abundant catalysts with high activity for the N_2_ reduction reaction. Here, it is reported that MnO particles act as a highly active catalyst for electrocatalytic hydrogenation of N_2_ to NH_3_ with excellent selectivity. In 0.1 m Na_2_SO_4_, this catalyst achieves a high Faradaic efficiency up to 8.02% and a NH_3_ yield of 1.11 × 10^−10^ mol s^−1^ cm^−2^ at −0.39 V versus reversible hydrogen electrode, with great electrochemical and structural stability. On the basis of density functional theory calculations, MnO (200) surface has a smaller adsorption energy toward N than that of H with the *N_2_ → *N_2_H transformation being the potential‐determining step in the nitrogen reduction reaction.

NH_3_ plays a key role in earth's ecosystem and is widely used as an activated nitrogen building block to manufacture fertilizers and other products.^1^, ^2^, ^3^ NH_3_ is also regarded as an attractive energy carrier with high energy density coupled with no CO_2_ emission.^4^, ^5^ The most abundant molecular N_2_, making up 78% of the atmosphere, is chemically inert, and does not engage in most chemical reactions due to the strong triple bond^6^ and the N_2_ to NH_3_ fixation is rather challenging. In N_2_‐fixing bacteria, nitrogenases biologically can catalyze the reduction of N_2_ to NH_3_ at ambient conditions.^7^, ^8^ High temperature and pressure however is involved in the Haber–Bosch process for industrial‐scale NH_3_ production.^6^, ^9^, ^10^, ^11^ This traditional process is not only energy‐intensive, but the H_2_ used as the feeding gas often comes from fossil fuels leading to serious greenhouse gas emission. In this context, a less energy‐demanding and environmentally benign alternative process for NH_3_ production is highly desirable.

Electrochemical reduction using proton from water as the hydrogen source can be powered by renewable energy from solar or wind sources under ambient reaction conditions, offering an attractive approach to convert N_2_ to NH_3_ in a green and sustainable manner.^12^, ^13^ Although tackling the energy‐ and H_2_‐intensive operations by the Haber–Bosch process, it is still challenged with N_2_ activation and electrocatalysts for N_2_ reduction reaction (NRR) are a prerequisite.^14^, ^15^ Efficient catalysts based on noble metals have been designed to perform NRR with remarkable catalytic performances.^16^, ^17^, ^18^, ^19^, ^20^, ^21^ An immediate outlook for large‐scale industrial applications points toward the use of systems not relying on expensive precious metals, which strongly encourages the development of non‐noble‐metal NRR electrocatalysts (Fe_2_O_3_‐CNT,^21^ PEBCD/C,^22^ NPC,^23^ defect‐rich MoS_2_ nanoflower,^24^ MoS_2_/CC,^25^ and Bi_4_V_2_O_11_/CeO_2_ 26).

As indispensable components of the cellular machinery, metal cations have various important biological functions, including in nucleic acids and protein structure stabilization to enzyme catalysis, signal transduction, photosynthesis,^27^, ^28^ etc. Photosynthesis is the principal energy converter on earth, and central to this process is photosystem II (PSII), a homodimeric multi‐subunit protein‐cofactor complex embedded in the thylakoid membrane.^29^, ^30^ PSII captures sunlight to power the most thermodynamically demanding reaction in biology: the photoinduced water oxidation to molecular O_2_, which is effectively catalyzed by a Mn‐containing cluster as the oxygen evolving center.^31^, ^32^ Inspired by this, a number of Mn‐based oxides have been developed for water oxidation electrocatalysis.^33^ Of note, Mn^2+^ is not involved in nitrogenases, but previous studies have suggested that Mn^2+^ can greatly enhance the catalytic activity of nitrogenases (N_2_ase) in extracts from the photosynthetic bacterium *Rhodospirillum rubrum*.^34^, ^35^ Although playing an important role in vitro activate N_2_ase for N_2_ fixation, Mn^2+^ is not required for the catalysis. In this regard, it is quite interesting to explore the electrochemical behavior of Mn oxide toward catalytic N_2_‐to‐NH_3_ fixation, which, however, has never been addressed before.

In this contribution, we demonstrate the first experimental verification that MnO particles on Ti mesh (MnO/TM) is a robust NRR catalyst for high‐performance electrohydrogenation of N_2_ to NH_3_ with excellent selectivity at ambient conditions. When tested in 0.1 m Na_2_SO_4_, this catalyst achieves a high Faradaic efficiency (FE) up to 8.02% and a large NH_3_ yield of 1.11 × 10^−10^ mol s^−1^ cm^−2^ at −0.39 V versus reversible hydrogen electrode (RHE). Density functional theory (DFT) calculations further reveal that the adsorption energy of N (ΔN*, −2.20 eV) is smaller than that of H (ΔH*, −1.56 eV) on MnO (200) surface and the *N_2_ → *N_2_H reaction is identified as the potential‐determining step of the NRR process and exhibits a low free energy change of 1.88 eV.

**Figure** **1**a,b shows X‐ray diffraction (XRD) patterns of MnCO_3_ and MnO scraped from TM. The peaks at 24.25°, 31.36°, 34.24°, 37.52°, 41.42°, 45.18°, 51.49°, and 51.69° are indexed to the (012), (104), (006), (110), (113), (202), (018), and (116) planes of MnCO_3_ (JCPDS No. 44‐1472), respectively. Other peaks are attributed to the KMn_8_O_16_ derived from the incomplete reduction of KMnO_4_ by glucose.^36^ The annealing product owns characteristic diffraction peaks at 34.96°, 40.59°, 58.74°, 70.22°, and 73.84° corresponding to the (111), (200), (220), (311), and (222) planes of MnO, respectively. Scanning electron microscopy (SEM) image of MnCO_3_/TM indicates the full coverage of TM (Figure S1, Supporting Information) with MnCO_3_ particles, as shown in Figure 1c. The SEM image (Figure 1d) of MnO/TM suggests the formation of roughed MnO particles with an average particle size of ≈0.65 µm (Figure 1d inset). The transmission electron microscopy (TEM) image (Figure 1e) further displays the MnO particles. High‐resolution TEM (HRTEM) image (Figure 1f) of MnO nanoparticle discloses clear lattices with an interplanar spacing of 0.221 nm, corresponding to the (200) plane of MnO crystal. Energy‐dispersive X‐ray (EDX) elemental mapping images (Figure 1g) manifest the uniform distribution of Mn and O elements in the MnO. Figure 1h,i presents the X‐ray photoelectron spectroscopy (XPS) spectra for MnO in Mn 2p and O 1s regions. For Mn 2p region, two diffraction peaks at 641.2 and 652.7 eV can be ascribed to the binding energies (BEs) of Mn 2p_3/2_ and Mn 2p_1/2_. The BE at 531.7 eV is assigned to the O 1s region with O^2−^ species.^37^ And the ratio of Mn:O is 0.99:1 obtained from XPS data, further validating the formation of MnO. Figure 1j shows the typical Raman spectrum of MnO at ≈318, 378, 481, 575, and 662 cm^−1^, which squares well with the XPS results.^38^ Electron paramagnetic resonance (EPR) spectrum of MnO (Figure S2, Supporting Information) shows pronounced signal with *g* = 2.045 that can be assigned to the unpaired electrons of the paramagnetic center of the Mn^2+^.^39^, ^40^ The pyrolysis process of MnCO_3_ was characterized by thermogravimetric analysis (TGA), as shown in Figure S3 (Supporting Information). MnCO_3_ starts to decompose at ≈255 °C, following a significant weight loss (≈37.5 wt%) at around 450 °C, which is slightly lower than the theoretical value of the weight loss for the decomposition of MnCO_3_ into MnO (≈38.26 wt%). It indicates that there should be carbon left in MnCO_3_ due to glucose decomposition. The carbon content was further determined by TGA analysis of MnO under an Ar‐air atmosphere. The first weight loss before 100 °C can be ascribed to the removal of adsorbed water, while the second weight loss in the temperature range of 375–700 °C is due to the decomposition of the amorphous carbon, and a slight increase of the weight in the temperature range from 700 to 900 °C is related to the oxidation of MnO into MnO*_x_* (Figure S4, Supporting Information).

**Figure 1:**
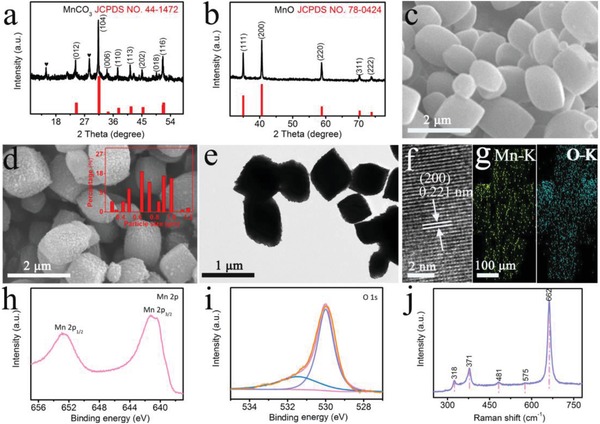
a,b) XRD patterns of MnCO_3_ and MnO. c,d) SEM images of MnCO_3_/TM and MnO/TM (inset: particle size distribution histogram of MnO). e) TEM and f) HRTEM images of MnO. g) EDX elemental mapping images of Mn and O elements of MnO. h,i) XPS spectra of MnO in the Mn 2p and O 1s regions. j) Raman spectrum of MnO.

The electrochemical measurements were performed in a two‐compartment electrochemical cell (Figure S5, Supporting Information) separated by a piece of Nafion membrane. A graphite rod, an Ag/AgCl electrode (filled with saturated KCl solution), and the prepared MnO/TM (with MnO loading of 0.85 mg) were used as counter electrode (Figure S6, Supporting Information), reference electrode, and working electrode, respectively. N_2_ gas was delivered into the cathodic compartment by N_2_ gas bubbling. All potentials are reported on the RHE scale. The produced NH_3_ was spectrophotometrically determined by the indophenol blue method,^41^ and the possible by‐product N_2_H_4_ was detected using the method of Watt and Chrisp.^42^ **Figure** **2**a shows the UV–vis absorption spectra of various electrolytes colored with indophenol indicator after electrocatalytic reaction for 3 h at different potentials ranging from −0.29 to −0.59 V (Figure S7, Supporting Information). Based on the calibration curves (Figure S8, Supporting Information), we determined the corresponding NH_3_ yields (Figure 2b) and FEs (Figure 2c). Furthermore, NH_3_ yields and FEs at various potentials (Figure S9, Supporting Information) determined by ion chromatography data (Table S1, Supporting Information) are quite comparable to the values obtained from the indophenol blue method. Both NH_3_ yields and FEs initially increase with potential being more negative. When the negative potential exceeds −0.39 V, the NH_3_ yields and FEs decrease obviously. These results might result from the competitive hydrogen‐evolving process, which can be suppressed in nonaqueous media to achieve higher current efficiency.^43^ Figure S10a (Supporting Information) exhibits the amount ofevolved H_2_ determined by gas chromatography from the headspace of the cell in N_2_‐saturated solution at various potentials. Figure S10b (Supporting Information) shows the selectivity of the catalyst toward H_2_ production at given potentials in N_2_‐saturated solutions. The unaccounted value may be attributed to the capacitance of the MnO/TM as well as dynamic H_2_ adsorption and desorption on MnO/TM.^44^ The mass ratio of H_2_/NH_3_ at various potentials is shown in Figure S10c (Supporting Information). At −0.39 V, MnO/TM affords the largest NH_3_ yield of 1.11 × 10^−10^ mol s^−1^ cm^−2^ and the highest FE of 8.02%, outperforming recent aqueous‐based NRR electrocatalysts, including Fe_2_O_3_‐CNT (3.58 × 10^−12^ mol s^−1^ cm^−2^, 0.15%),^21^ PEBCD/C (2.58 × 10^−11^ mol s^−1^ cm^−2^, 2.85%),^22^ MoS_2_/CC (8.08 × 10^−11^ mol s^−1^ cm^−2^, 1.17%),^25^ Mo_2_N (4.60 × 10^−10^ mol s^−1^ cm^−2^, 4.5%),^45^ MoO_3_ (4.80 × 10^−10^ mol s^−1^ cm^−2^, 1.9%),^46^ MoN NA/CC (3.01 × 10^−10^ mol s^−1^ cm^−2^, 1.15%),^47^ Fe_3_O_4_/Ti (5.6 × 10^−11^ mol s^−1^ cm^−2^, 2.6%),^48^ TiO_2_/Ti (9.16 × 10^−11^ mol s^−1^ cm^−2^, 2.5%),^49^ VN/TM (8.40 × 10^−11^ mol s^−1^ cm^−2^, 2.25%),^50^ hollow Cr_2_O_3_ microspheres (25.3 µg h^−1^ mg^−1^_cat._, 6.78%),^51^ and TiO_2_‐rGO (15.13 µg h^−1^ mg^−1^_cat._, 3.3%).^52^ A more detailed comparison is listed in Table S2 (Supporting Information).

**Figure 2:**
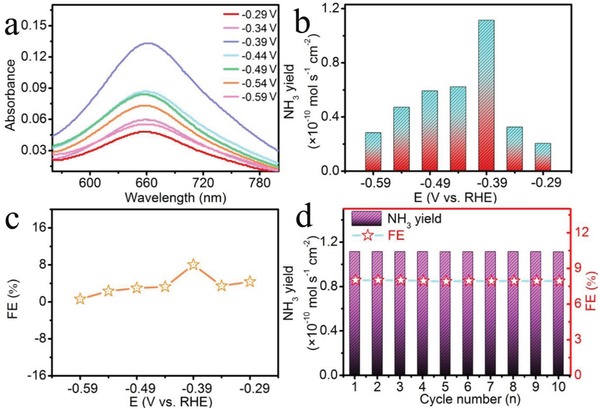
a) UV–vis absorption spectra of the electrolytes stained with indophenol indicator after NRR electrolysis at a series of potentials for 3 h. b) NH_3_ yields and c) FEs for MnO/TM at a series of potentials. d) Cycling test of MnO/TM at a potential of −0.39 V. The data were subtracted by the absorbance intensities of electrolytes after NRR electrolysis in Ar.

We compared the catalytic NRR performances of blank TM, MnCO_3_/TM, and MnO/TM. Figure S11 (Supporting Information) shows the NH_3_ yields for three electrodes after 3 h electrolysis at −0.39 V. Obviously, blank TM shows very poor NRR activity. Of note, although MnCO_3_/TM is also active for NRR, it only achieves a much lower NH_3_ yield compared with MnO/TM. The electrochemical impedance spectroscopy (EIS) data (Figure S12, Supporting Information) also suggest that MnO/TM has a lower charge transfer resistance^53^ than MnCO_3_/TM, leading faster NRR kinetics. For the case of NH_3_ electrosynthesis, the by‐product N_2_H_4_ seems to be easy to generate. However, there is no N_2_H_4_ detected in our system (Figure S13, Supporting Information), revealing the excellent selectivity of MnO/TM for NH_3_ formation. Stability of the MnO/TM catalyst for electrochemical NRR was assessed by consecutive electrolysis at −0.39 V. As observed in Figure 2d, MnO/TM has negligible change in NH_3_ yields and FEs during the cycling tests for ten times, implying its strong electrochemical stability.

To probe the durability, we collected the time‐dependent current density curve of MnO/TM at −0.39 V. As shown in **Figure** **3**a, this catalyst can maintain its catalytic activity for at least 25 h. After NRR durability test, this catalyst still maintains its particle morphology (Figure 3b) and is also MnO in nature (Figure 3c,d and Figure S14, Supporting Information). The particle size of MnO is distributed with an average size of ≈0.65 µm (Figure 3b inset).

**Figure 3:**
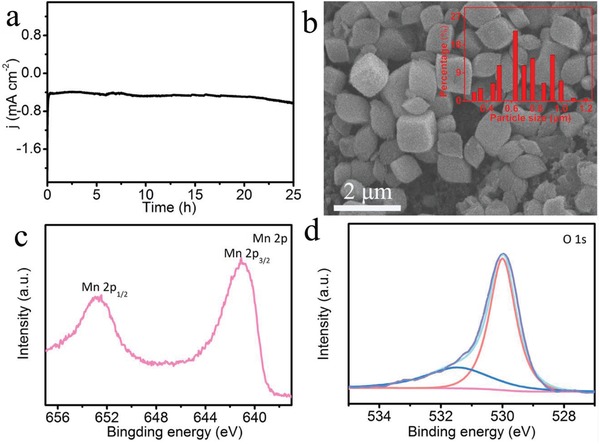
a) Time‐dependent current density curve of MnO/TM at potential of −0.39 V. SEM image of b) MnO/TM (inset: particle size distribution histogram of MnO) and XPS spectra of MnO in the c) Mn 2p and d) O 1s after durability test.

We also performed NRR experiments for MnO/TM under N_2_ atmosphere at an open‐circuit or under Ar gas at −0.39 V for 3 h. As shown in Figure S15 (Supporting Information), no apparent NH_3_ was detected for both cases using the indophenol blue method, indicating NH_3_ was produced by N_2_ reduction in the presence of MnO catalyst. The formation of NH_4_^+^ during the electrochemical NRR experiments was also validated by Raman spectroscopy under 785 nm laser excitation (Figure S16, Supporting Information). Given that Na_2_SO_4_ could affect the Raman features of NH_4_^+^, we compared the Raman spectrum of NRR sample collected from the electrochemical experiment at −0.39 V with those of 0.1 m Na_2_SO_4_ and 0.1 m Na_2_SO_4_ + NH_4_^+^. At 3200 cm^−1^, there is no peak for 0.1 m Na_2_SO_4_, while NRR sample and 0.1 m Na_2_SO_4_ + NH_4_^+^ have intensive peaks, indicating the generation of NH_4_^+^ in solution during the electrolysis.^54^, ^55^ We further performed ^15^N isotopic labeling experiment to verify the N source of the NH_3_ produced, using a doublet coupling for ^15^NH_4_^+^ standard sample as reference. Figure S17 (Supporting Information) shows the ^1^H nuclear magnetic resonance (^1^H NMR) spectra. As observed, supplying Ar to the electrolysis system failed to produce NH_4_^+^ in the electrolyte; however, ^15^NH_4_^+^ was detected when ^15^N_2_ was fed. These observations also confirm that the NH_3_ in the electrolyte was indeed generated via electrocatalytic N_2_ reduction by MnO.

For appreciable buildup of NH*_x_* intermediates, an ideal NRR catalyst would require a high selectivity of N_2_ adsorption with respect to H, that is, the adsorption energy of N (*E*_ads_ N*) is smaller than that of H (*E*_ads_ H*) on the catalyst surface.^56^, ^57^ Our DFT calculations demonstrate that the adsorption of N on Mn atom and H on O atom of the MnO (200) surface yield the largest adsorption energy of −2.20 and −1.56 eV, respectively, which indicates that our catalyst has higher selectivity for N.

In general, the hydrogenation of N_2_ involves supplying hydrogen atoms to N_2_ one‐by‐one from the electrolyte and gaining electrons from the electrode surface. The standard hydrogen electrode (SHE) free energy profile (**Figure** **4**a) shows that the reaction from *N_2_ to *NNH with an uphill free energy change of 1.88 eV is the potential‐determining step of the NRR process on the MnO (200) surface. At the initial stage (Figure S18, Supporting Information), N_2_ is physically adsorbed on the top of Mn atom with N≡N and N—Mn bond lengths of 1.12 and 2.73 Å, respectively. Subsequently, the N≡N bond is elongated to 1.21 Å while the N—Mn bond is shortened to 2.05 Å due to that the N≡N dimer is hydrogenated to *NNH, as shown in Figure S18 (Supporting Information). As displayed in Figure 4b, the atom‐projected density of state (DOS) for the *N_2_ and *NNH structures is substantially from the substrate MnO. The zooming‐in DOS of the N_2_/MnO configuration shows that the two N atoms are completely overlapping at 1.0–1.5 eV above the Fermi level, suggesting the intact feature of N_2_ molecule and the weak interactions between N_2_ and MnO surface. As for the structure of NNH adsorbed on MnO, the enlarged DOS image evidences the hybridization of N2 with H and N1 atoms, as well as the bonding interactions between N1 atom and the substrate around −1.5 to −1.0 eV in the valence band. Consequently, the N‐2p states locating above the Fermi level are stretched from about 0.25 to 1.5 eV and becoming less overlapping with respect to the N_2_ portion in the DOS of N_2_/MnO, indicating the N_2_ molecules are activated by the added H atom. Furthermore, the charge density difference, defined as △ρ = ρ(*A) − ρ(*) − ρ(A) (A = adsorbate), of *N_2_ and *NNH states are visualized in Figure 4c. Because of the weak vdW interactions between N_2_ and the substrate, the electron transfer between them is greatly limited. However, it is much more impressive for the *NNH configuration, where the depletion of electrons occurs in the region between the two N atoms while the accumulation of electrons occurs on the N—Mn and N—H bonds (Figure 4c). This indicates the remarkable dissociation of the N—N bonds in the first hydrogenation step. Bader charge analysis^58^ manifests that the lower N atom obtains 0.13 electrons from the coordinated Mn atom, and the upper N atom grabs 0.38 electrons from the hydrogen atom (Figure 4c).

**Figure 4:**
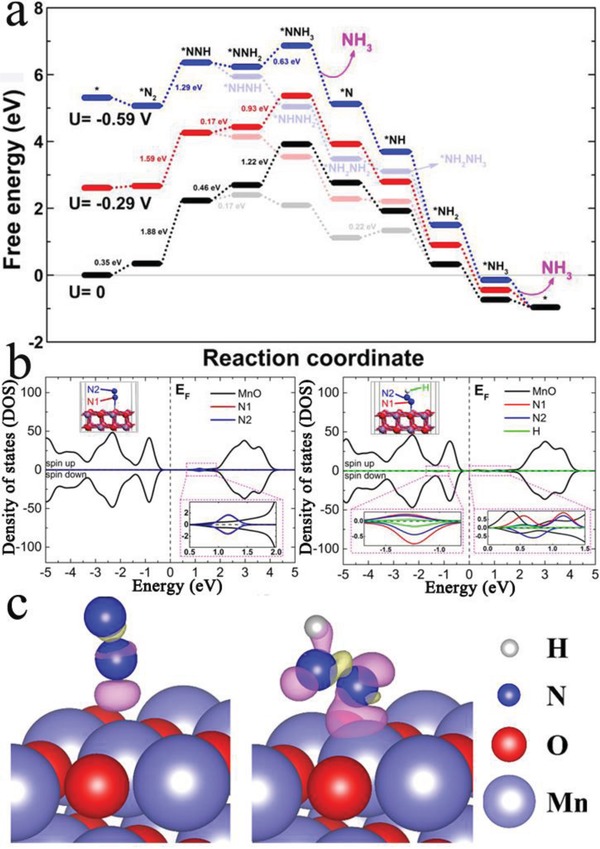
a) Free energy profile of NRR process on MnO (200) surface. An asterisk (*) denotes the adsorption site. The competitive processes are shown in light colors. b) DOS of *N_2_ and *NNH. c) Charge density difference of *N_2_ and *NNH. The electron excess and deficiency are displayed as purple and yellow isosurfaces, respectively. The level of isosurface is 0.001 and 0.003 e Å^−1^ for *N_2_ and *NNH, respectively.

The addition of the second hydrogen atom is 0.46 or 0.17 eV uphill to form *NNH_2_ or *NHNH on the SHE free energy profile. Obviously, the formation of *NHNH is an energetically preferable hydrogenation step. This pathway is even more feasible to add the third hydrogen atom, since the formation of *NHNH_2_ is −0.31 eV downhill, but an uphill free energy of 1.22 eV is required to form *NNH_3_. After that, all the following hydrogen steps are downhill in free energy (exergonic), except for a small uphill energy change of 0.22 eV from *NH_2_NH_2_ to *NH_2_NH_3_. This value is much smaller than the desorption energy of 0.60 eV for NH_2_NH_2_ to escape from the MnO (200) surface. Therefore, the formation of N_2_H_4_ as a by‐product is prohibited.

In order to better interpret the NRR process under the experimental condition, the effect of potential of −0.29 or −0.59 V on the reactions is involved in the calculations. As compared in Figure 4a, the reaction free energy of the determining step on the SHE energy profile is reduced to 1.59 or 1.29 eV with the applied potential of −0.29 or −0.59 V, respectively. The uphill energy change is eliminated for the reaction step from *NNH to *NHNH (or *NNH_2_), and for the following reaction steps. The large energy change of the next step on the competitive reaction process is also reduced to 0.93 or 0.63 eV. The decline and elimination of the uphill reaction steps suggest the beneficial effect of the applied potentials on the hydrogenation process of N_2_. The energy profile of the NRR process under the potential of −0.39 V accompanying with the values of the standard reaction (0 V) is presented in Figure S19 (Supporting Information), in order to reduce spatial redundancy of Figure 4a. The potential of −0.39 V makes moderate efforts to adjust the uphill energy change on the reaction pathway, as compared with the results of −0.29 and −0.59 V. Remarkably, although the adsorption of N_2_ and NNH species is more stable on the MnCO_3_ (104) surface, the free energy difference of the critical step *N_2_ → *NNH on the MnCO_3_ (104) is 1.98 eV, which is larger than the value of 1.88 eV for the reaction on the MnO (200) surface (Table S3, Supporting Information). Such smaller free energy difference of *N_2_ → *NNH and the lower charge transfer resistance (Figure S12, Supporting Information) contribute to the higher NRR activity for MnO/TM.

In summary, MnO has been experimentally proven as a high‐performance and durable catalyst for ambient electrohydrogenation of N_2_ to NH_3_ in neutral media. This catalyst achieves superior performances in current efficiency and NH_3_ yield of 8.02% and 1.11 × 10^−10^ mol s^−1^ cm^−2^ at −0.39 V, respectively. Theoretical calculations further reveal the preferential adsorption of N atoms compared to H atoms on the catalyst and the potential‐determining step of *N_2_ → *N_2_H reaction in the NRR process. Constructing carbon‐based nanohybrids with enhanced conductivity is a promising way to further enhance the NRR performances of Mn oxide catalysts,^59^ and engineering surface oxygen vacancies for more efficient molecular N_2_ adsorption and activation may also provide another viable avenue.^26^, ^60^, ^61^

## Experimental Section

*Materials*: KMnO_4_, glucose, salicylic acid, sodium citrate, sodium hypochlorite (NaClO), Na_2_[Fe(CN)_5_NO] · 2H_2_O, and sodium nitroferricyanide (C_5_FeN_6_Na_2_O) were purchased from Sigma‐Aldrich Chemical Reagent Co., Ltd. Sodium sulfate (Na_2_SO_4_), isopropyl alcohol, ethanol, and ^15^N_2_ gas were purchased from Aladdin Ltd. (Shanghai, China). 211 Nafion membrane (Dupont) and TM were provided by Hongshan District, Wuhan Instrument Surgical Instruments business. The ultrapure water used throughout all experiments was purified through a Millipore system.

*Preparation of MnCO_3_/TM and MnO/TM*: To prepare MnCO_3_/TM, 0.8 g of KMnO_4_, 1.0 g of glucose, and 45 mL of deionized water were mixed with magnetic stirring. After stirring for 30 min, the mixture was transferred and sealed in a 50 mL Teflon‐lined autoclave with a piece of TM, then heated at 180 °C for 10 h, and finally cooled to room temperature. The obtained material was collected by centrifuge, washed alternately with deionized water and ethanol for three times, and dried in vacuum oven at 80 °C overnight. MnO/TM material was prepared by heating the resulting MnCO_3_/TM at 550 °C for 4 h in Ar.

*Characterizations*: XRD patterns were obtained from a Shimazu XRD‐6100 diffractometer working with Cu Kα radiation (40 kV, 30 mA) of wavelength 0.154 nm (Shimadzu, Japan). SEM images were collected from the tungsten lamp‐equipped SU3500 scanning electron microscope at an accelerating voltage of 20 kV (Hitachi, Japan). TEM images were obtained from a Zeiss Libra 200FE transmission electron microscope operated at 200 kV. XPS measurements were performed on an ESCALABMK II X‐ray photoelectron spectrometer using Mg as the exciting source. The absorbance data of spectrophotometer were measured on UV–vis spectrophotometer. TGA were performed on a Perkin‐Elmer Model Pyris1 TGA apparatus at a heating rate of 10 °C min^−1^ in flowing Ar or Ar‐air mixture. The Raman spectra were collected on a Renishaw InVia Raman spectrometer under a backscattering geometry (λ = 532 nm). EPR spectra were obtained from the Bruker EMX‐10/12 variable‐temperature apparatus. A gas chromatograph (Shimadzu, GC‐2014C) equipped with MolSieve 5A column was used for H_2_ quantifications. Gas‐phase product was sampled every 1000 s using a gas‐tight syringe (Hamilton). The ion chromatography was conducted on Swiss Wangtong ECO. ^1^H NMR spectra were recorded on a Bruker AVANCE III 500 HD spectrometer with a 5 mm BBFO smart probe, operating at 500.13 MHz. ^1^H NMR experiments were carried out at 303 K for 5% w/v sample solution in DMSO‐d6. The spectral windows were set to 12.5 kHz (25 ppm), a total of 16 scans were recorded, a π/2 pulse length of 11.6 µs, and 64 K data points with 3 s recycle delay for each sample. Topspin software version is 3.5 pl6. All ^1^H chemical shifts are referenced to the resonances of DSS standard (δ = 0.00).

*Electrochemical N_2_ Reduction Measurements*: All experiments were carried out at room temperature. The potentials reported in this work were converted to RHE scale via calibration with the following equation: *E* (vs RHE) = *E* (vs Ag/AgCl) + pH × 0.059 V and the presented current densities were normalized to the geometric surface area. For N_2_ reduction experiments, the Na_2_SO_4_ electrolyte was purged with N_2_ for 30 min before the measurement. Potentiostatic tests were conducted in N_2_‐saturated 0.1 m Na_2_SO_4_ solution (30 mL) in a two‐compartment cell, which was separated by Nafion 211 membrane. For comparison, potentiostatic test in Ar‐saturated 0.1 m Na_2_SO_4_ solution was also conducted in this work. The concentration of synthesized NH_3_ was calculated after subtracting that in Ar.

*Determination of NH_3_*: The produced NH_3_ was detected with indophenol blue by UV–vis absorption spectra. In detail, 4.0 mL electrolyte was removed from the cathodic chamber and added into 50 µL oxidizing solution containing NaClO (ρ_Cl_ = 4–4.9) and NaOH (0.75 m), followed by adding 500 µL coloring solution containing 0.4 m C_7_H_6_O_3_ and 0.32 m NaOH, and 50 µL catalyst solution (0.1 g Na_2_[Fe(CN)_5_NO] · 2H_2_O diluted to 10 mL with deionized water) in turn. Absorbance measurements were performed after 2 h at λ = 655 nm. The concentration–absorbance curve was calibrated using standard NH_4_Cl solution with NH_4_^+^ concentrations of 0.0, 0.1, 0.2, 0.3, 0.4, and 0.5 µg mL^−1^ in 0.1 m Na_2_SO_4_. The fitting curve (*y* = 0.7036*x* + 0.029, *R* ^2^ = 0.999) shows good linear relation of absorbance value with NH_3_ concentration by three times independent calibrations.

*Determination of N_2_H_4_*: N_2_H_4_ presented in the electrolyte was determined by the method of Watt and Chrisp. The p‐C_9_H_11_NO (5.99 g), HCl (30 mL), and C_2_H_5_OH (300 mL) were mixed and the resulting mixture was used as a color reagent. In detail, 5 mL electrolyte was removed from the electrochemical reaction vessel, and added into 5 mL prepared color reagent at 25 °C. The obtained calibration curve of N_2_H_4_ is *y* = 0.433*x* + 0.044 with *R* ^2^ = 0.999.

*Determination of NH_3_ Yield Rate and the Calculation of FE*: NH_3_ yield rate was calculated using the following equation

(1) $${\mathrm{NH}}_{3}\;\mathrm{yield}\;\mathrm{rate}=\left[{\mathrm{NH}}_{3}\right]\times V/\left(17\times t\times A\right)$$

The FE was calculated according to the following equation

(2) $$\mathrm{FE}=3\times F\times \left[{\mathrm{NH}}_{3}\right]\times V/\left(17\times Q\right)$$

where [NH_3_] is the measured NH_3_ concentration; *V* is the volume of the cathodic reaction for NH_3_ collection; *t* is the potential applied time; *A* is the geometric area; *m* is the loaded mass of catalyst; *F* is the Faraday constant; and *Q* is the quantity of applied electricity.

*DFT Calculation Details*: Spin‐polarized DFT+*U* calculations were performed using the Vienna ab initio simulation package (VASP)^62^ to improve the description of the on‐site Coulomb interactions between Mn (3d) electrons, where the value of *U*_eff_ = *U* − *J* was set to 4.0 eV.^63^, ^64^ The projector augmented wave (PAW) potentials^65^ were used for the treatment of core electrons. The generalized gradient approximation (GGA) with the Perdew–Burke–Ernzerhof (PBE) functional^66^ was applied to describe the electron exchange correlation interactions. In order to investigate the key steps of the NRR on the MnO (200) surface, the calculations were performed based on a model of 2 × 2 × 1 supercell, with a vacuum of 20 Å added in the *z*‐direction. The energy cutoff of the plane‐wave basis sets was set to 450 eV. The ionic relaxation was performed until the force on each atom converge to within 0.01 eV Å^−1^. The K points were sampled with 5 × 5 × 1 by Monkhorst–Pack method.^67^ The magnetic ordering of the Mn atoms in the slab was arranged in type‐II antiferromagnetic, as suggested by Pask et al.^68^ Under the SHE condition, the reaction free energies of the NRR steps were calculated as:^69^ *G* = *E*_DFT_ + *E*_ZPE_ − *T*Δ*S*, where *E*_DFT_ is the DFT calculated energy, *E*_ZPE_ and *T*Δ*S* are obtained by DFT vibration frequency calculations, and presented in Table S4 (Supporting Information). In order to consider the effect of an applied electric potential on the electrode reaction, a value of −*neU* was added to calculate the free energy of each step, where *n* is the number of electrons involved in the reaction, and *U* is the applied bias.^69^

## Conflict of Interest

The authors declare no conflict of interest.

## Supporting information

Supplementary

## References

[1] R. Schlögl , Angew. Chem., Int. Ed. 2003, 42, 2004. 10.1002/anie.200301553 12746811

[2] T. Murakami , T. Nishikiori , T. Nohira , Y. Ito , J. Am. Chem. Soc. 2003, 125, 334. 12517136 10.1021/ja028891t

[3] V. Rosca , M. Duca , M. T. de Groot , M. T. Koper , Chem. Rev. 2009, 109, 2209. 19438198 10.1021/cr8003696

[4] C. J. Pickett , J. Talarmin , Nature 1985, 317, 652.

[5] A. Klerke , C. H. Christensen , J. K. Norskov , T. Vegge , J. Mater. Chem. 2008, 18, 2304.

[6] V. Smil , Sci. Am. 1997, 277, 76.

[7] B. K. Burgess , D. J. Lowe , Chem. Rev. 1996, 96, 2983. 11848849 10.1021/cr950055x

[8] B. M. Hoffman , D. Lukoyanov , Z. Yang , D. Dean , L. C. Seefeldt , Chem. Rev. 2014, 114, 4041. 24467365 10.1021/cr400641x PMC4012840

[9] J. R. Jennings , Catalytic Ammonia Synthesis: Fundamentals and Practice, Plenum, New York 1991.

[10] S. Li , D. Bao , M. Shi , B. Wulan , J. Yan , Q. Jiang , Adv. Mater. 2017, 29, 1700001. 10.1002/adma.201700001 28681965

[11] D. Bao , Q. Zhang , F. Meng , H. Zhong , M. Shi , Y. Zhang , J. Yan , Q. Jiang , X. Zhang , Adv. Mater. 2017, 29, 1604799. 10.1002/adma.201604799 27859722

[12] M. A. Shipman , M. D. Symes , Catal. Today 2017, 286, 57.

[13] V. Kyriakou , I. Garagounis , E. Vasileiou , A. Vourros , M. Stoukides , Catal. Today 2017, 286, 2.

[14] Z. W. Seh , J. Kibsgaard , C. F. Dickens , I. Chorkendorff , J. K. Nørskov , T. F. Jaramillo , Science 2017, 355, eaad4998. 28082532 10.1126/science.aad4998

[15] C. Guo , J. Ran , A. Vasileff , S. Qiao , Energy Environ. Sci. 2018, 11, 45.

[16] M. Shi , D. Bao , B. R. Wulan , Y. Li , Y. Zhang , J. Yan , Q. Jiang , Adv. Mater. 2017, 29, 1606550. 10.1002/adma.201606550 28240391

[17] Z. Wang , Y. Li , H. Yu , Y. Yu , H. Xue , X. Li , H. Wang , L. Wang , ChemSusChem 2018, 11, 3480. 30109915 10.1002/cssc.201801444

[18] M. Nazemi , S. R. Panikkanvalappil , M. A. El‐Sayed , Nano Energy 2018, 49, 316.

[19] H. Liu , S. H. Han , Y. Zhao , Y. Zhu , X. Tian , J. Zeng , Y. Chen , J. Mater. Chem. A 2018, 6, 3211.

[20] K. Kugler , M. Luhn , J. A. Schramm , K. Rahimi , M. Wessling , Phys. Chem. Chem. Phys. 2015, 17, 3768. 25556769 10.1039/c4cp05501b

[21] S. Chen , S. Perathoner , C. Ampelli , C. Mebrahtu , D. Su , G. Centi , Angew. Chem., Int. Ed. 2017, 56, 2699. 10.1002/anie.201609533 28128489

[22] G. Chen , X. Cao , S. Wu , X. Zeng , L. Ding , M. Zhu , H. Wang , J. Am. Chem. Soc. 2017, 139, 9771. 28693318 10.1021/jacs.7b04393

[23] Y. Liu , Y. Su , X. Quan , X. Fan , S. Chen , H. Yu , H. Zhao , Y. Zhang , J. Zhao , ACS Catal. 2018, 8, 1186.

[24] X. Li , T. Li , Y. Ma , Q. Wei , W. Qiu , H. Guo , X. Shi , P. Zhang , A. M. Asiri , L. Chen , B. Tang , X. Sun , Adv. Energy Mater. 2018, 8, 201801357.

[25] L. Zhang , X. Ji , X. Ren , Y. Ma , X. Shi , Z. Tian , A. M. Abdullah , L. Chen , B. Tang , X. Sun . Adv. Mater. 2018, 30, 1800191. 10.1002/adma.201800191 29808517

[26] C. Lv , C. Yan , G. Chen , Y. Ding , J. Sun , Y. Zhou , G. Yu , Angew. Chem., Int. Ed. 2018, 57, 6073. 10.1002/anie.201801538 29473991

[27] L. A. Finney , T. V. O'Halloran , Science 2003, 300, 931. 12738850 10.1126/science.1085049

[28] T. Dudev , C. Lim , Chem. Rev. 2014, 114, 538. 24040963 10.1021/cr4004665

[29] B. Ke , Photosynthesis: Photobiochemistry and Photobiophysics, Kluwer Academic, Dordrecht, The Netherlands 2001.

[30] B. Kok , B. Forbush , M. Gloin , Photochem. Photobiol. 1970, 11, 457. 5456273 10.1111/j.1751-1097.1970.tb06017.x

[31] K. N. Ferreira , T. M. Iverson , K. Maghlaoui , J. Barber , S. Iwata , Science 2004, 303, 1831. 14764885 10.1126/science.1093087

[32] B. Loll , J. Kern , W. Saenger , A. Zouni , J. Biesiadka , Nature 2005, 438, 1040. 16355230 10.1038/nature04224

[33] B. M. Hunter , H. B. Gray , A. D. Müller , Chem. Rev. 2016, 116, 14120. 27797490 10.1021/acs.chemrev.6b00398

[34] P. W. Ludden , R. H. Burris . Science 1976, 194, 424. 824729 10.1126/science.824729

[35] S. Nordlund , U. Erikason , H. Baltscheffsky , Biochim. Biophys. Acta 1977, 462, 187. 410446 10.1016/0005-2728(77)90201-8

[36] G. Xu , Y. Xu , J. Fang , F. Fu , H. Sun , L. Huang , S. Yang , S. Sun , ACS Appl. Mater. Interfaces 2013, 5, 6316. 23758592 10.1021/am401355w

[37] Y. Sun , X. Hu , W. Luo , Y. Huang , J. Mater. Chem. 2012, 22, 19190.

[38] K. Ramesh , L. Chen , F. Chen , Y. Liu , Z. Wang , Y. Han , Catal. Today 2008, 131, 477.

[39] T. Chen , P. Huo , J. Hou , J. Xu , Q. Zhu , J. Dai , Inorg. Chem. 2016, 55, 12758. 27989159 10.1021/acs.inorgchem.6b02062

[40] P. A. G. Beermann , B. R. McGarvey , S. Muralidharan , R. C. Sung , Chem. Mater. 2004, 16, 915.

[41] W. Qiu , X. Xie , J. Qiu , W. Fang , R. Liang , X. Ren , X. Ji , G. Cui , A. M. Asiri , G. Cui , B. Tang , X. Sun , Nat. Commun. 2018, 9, 3485. 30154483 10.1038/s41467-018-05758-5 PMC6113289

[42] G. W. Watt , J. D. Chrisp , Anal. Chem. 1952, 24, 2006.

[43] F. Zhou , L. M. Azofra , M. Ali , M. Kar , A. N. Simonov , C. McDonnell‐Worth , D. R. MacFarlane , Energy Environ. Sci. 2017, 10, 2516.

[44] J. Wang , L. Yu , L. Hu , G. Chen , H. Xin , X. Feng , Nat. Commun. 2018, 9, 1795. 29765053 10.1038/s41467-018-04213-9 PMC5953946

[45] X. Ren , G. Cui , L. Chen , F. Xie , Q. Wei , Z. Tian , X. Sun , Chem. Commun. 2018, 54, 8474. 10.1039/c8cc03627f 30003198

[46] J. Han , X. Ji , X. Ren , G. Cui , L. Li , F. Xie , H. Wang , B. Li , X. Sun , J. Mater. Chem. A 2018, 6, 12974.

[47] L. Zhang , X. Ji , X. Ren , Y. Luo , X. Shi , A. M. Asiri , B. Zheng , X. Sun , ACS Sustainable Chem. Eng. 2018, 6, 9550.

[48] Q. Liu , X. Zhang , B. Zhang , Y. Luo , G. Cui , F. Xie , X. Sun , Nanoscale 2018, 10, 14386. 30027985 10.1039/c8nr04524k

[49] R. Zhang , X. Ren , X. Shi , F. Xie , B. Zheng , X. Guo , X. Sun , ACS Appl. Mater. Interfaces 2018, 10, 28251. 30117725 10.1021/acsami.8b06647

[50] R. Zhang , Y. Zhang , X. Ren , G. Cui , A. M. Asiri , B. Zheng , X. Sun , ACS Sustainable Chem. Eng. 2018, 6, 9545.

[51] Y. Zhang , W. Qiu , Y. Ma , Y. Luo , Z. Tian , G. Cui , F. Xie , L. Chen , T. Li , X. Sun , ACS Catal. 2018, 8, 8540.

[52] X. Zhang , Q. Liu , X. Shi , A. M. Asiri , Y. Luo , T. Li , X. Sun , J. Mater. Chem. A 2018, 6, 17303.

[53] Y. Ji , L. Yang , X. Ren , G. Cui , X. Xiong , X. Sun , ACS Sustainable Chem. Eng. 2018, 6, 11186.

[54] P. Vítek , H. G. M. Edwards , J. Jehlicka , C. Ascaso , A. De Los Ríos , S. Valea , S. E. Jorge‐Villar , A. F. Davila , J. Wierzchos , Philos. Trans. R. Soc. A 2010, 368, 3205. 10.1098/rsta.2010.0059 20529955

[55] R. L. Carter , Spectrochim. Acta, Part A 2002, 58, 3185. 10.1016/s1386-1425(02)00104-x 12511103

[56] A. R. Singh , B. A. Rohr , J. A. Schwalbe , M. Cargnello , K. Chan , T. F. Jaramillo , J. K. Nørskov , ACS Catal. 2017, 7, 706.

[57] T. Oshikiri , K. Ueno , H. Misawa , Angew. Chem., Int. Ed. 2016, 55, 3942. 10.1002/anie.201511189 26890286

[58] R. F. W. Bader , Atoms in Molecules: A Quantum Theory, Oxford University Press, Oxford 1990.

[59] Y. Wang , F. Gong , X. Wu , X. Shi , A. M. Asiri , T. Li , Q. Liu , X. Sun , unpublished.

[60] C. Li , T. Wang , Z. Zhao , W. Yang , J. Li , A. Li , Z. Yang , G. A. Ozin , J. Gong , Angew. Chem., Int. Ed. 2018, 57, 5278. 10.1002/anie.201713229 29457861

[61] H. Hirakawa , M. Hashimoto , Y. Shiraishi , T. Hirai , J. Am. Chem. Soc. 2017, 139, 10929. 28712297 10.1021/jacs.7b06634

[62] M. D. Segall , J. D. L. Philip , M. J. Probert , C. J. Pickard , P. J. Hasnip , S. J. Clark , M. C. Payne , J. Phys. Condens. Matter 2002, 14, 2717.

[63] A. Jain , G. Hautier , S. P. Ong , C. J. Moore , C. C. Fischer , K. A. Persson , G. Ceder , Phys. Rev. B 2011, 84, 045115.

[64] L. Wang , T. Maxisch , G. Ceder , Phys. Rev. B 2006, 73, 195107.

[65] P. E. Blochl , Phys. Rev. B 1994, 50, 17953. 10.1103/physrevb.50.17953 9976227

[66] J. P. Perdew , J. A. Chevary , S. H. Vosko , K. A. Jackson , M. R. Pederson , D. J. Singh , C. Fiolhais , Phys. Rev. B 1992, 46, 6671. 10.1103/physrevb.46.6671 10002368

[67] H. J. Monkhorst , J. D. Pack , Phys. Rev. B 1976, 13, 5188.

[68] J. Pask , D. Singh , I. Mazin , C. Hellberg , J. Kortus , Phys. Rev. B 2001, 64, 024403.

[69] E. Skulason , T. Bligaard , S. Gudmundsdóttir , F. Studt , J. Rossmeisl , F. Abild‐Pedersen , J. K. Nørskov , Phys. Chem. Chem. Phys. 2012, 14, 1235. 22146855 10.1039/c1cp22271f

