# Supplementary material for: Electrocatalytic Hydrogenation of N2 to NH3 by MnO: Experimental and Theoretical Investigations
Source: Adv Sci (Weinh). 2018 Nov 9;6(1):1801182. doi: 10.1002/advs.201801182 (PMC6325594; doi:10.1002/advs.201801182)
Supplement: Supplementary file 1 — Supplementary [file ADVS-6-1801182-s001.pdf]

Copyright WILEY-VCH Verlag GmbH & Co. KGaA, 69469 Weinheim, Germany, 2018.

ADVANCED SCIENCE
Open Access

Supporting Information

for *Adv. Sci.,* DOI: 10.1002/advs.201801182

Electrocatalytic Hydrogenation of N_2 to NH_3 by MnO: Experimental and Theoretical Investigations

Zao Wang, Feng Gong, Ling Zhang, Rui Wang, Lei Ji, Qian Liu, Yonglan Luo, Haoran Guo, Yuehui Li, Peng Gao, Xifeng Shi, Baihai Li, Bo Tang,* and Xuping Sun**

Supporting Information

Electrocatalytic Hydrogenation of N_2 to NH_3 by MnO: Experimental and Theoretical Investigations

Zao Wang, Feng Gong, Ling Zhang, Rui Wang, Lei Ji, Qian Liu, Yonglan Luo, Haoran Guo, Yuehui Li, Peng Gao, Xifeng Shi, Baihai Li,* Bo Tang,* and Xuping Sun*

Z. Wang, L. Zhang, R. Wang, L. Ji, Y. Luo, Prof. X. Sun
Institute of Fundamental and Frontier Sciences, University of Electronic Science and Technology of China, Chengdu 610054, Sichuan (China)
E-mail: xpsun@uestc.edu.cn
Z. Wang, L. Zhang, L. Ji
College of Chemistry, Sichuan University, Chengdu 610064, Sichuan (China)
Dr. F. Gong, Dr. Q. Liu, H. Guo, Prof. B. Li
School of Materials and Energy, University of Electronic Science and Technology of China, Chengdu 611731, Sichuan (China)
E-mail: libaihai@uestc.edu.cn
Y. Li, Dr. P. Gao
International Center for Quantum Materials and Electron Microscopy Laboratory, School of Physics, Peking University, Beijing, 100871, (China)
Collaborative Innovation Centre of Quantum Matter, Beijing 100871, (China)
Dr. X. Shi, Prof. B. Tang
College of Chemistry, Chemical Engineering and Materials Science, Shandong Normal University, Jinan 250014, Shandong (China)
Email: tangb@sdnu.edu.cn
Z. Wang and F. Gong contributed equally to this work.

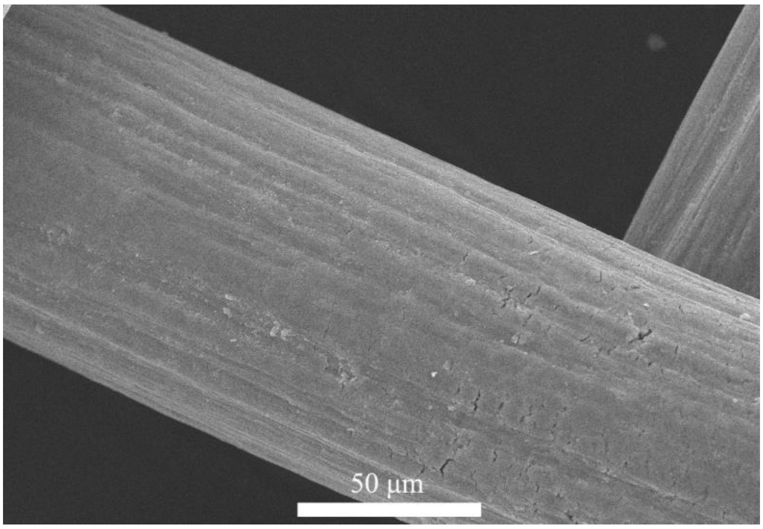

Figure S1. SEM image of TM.

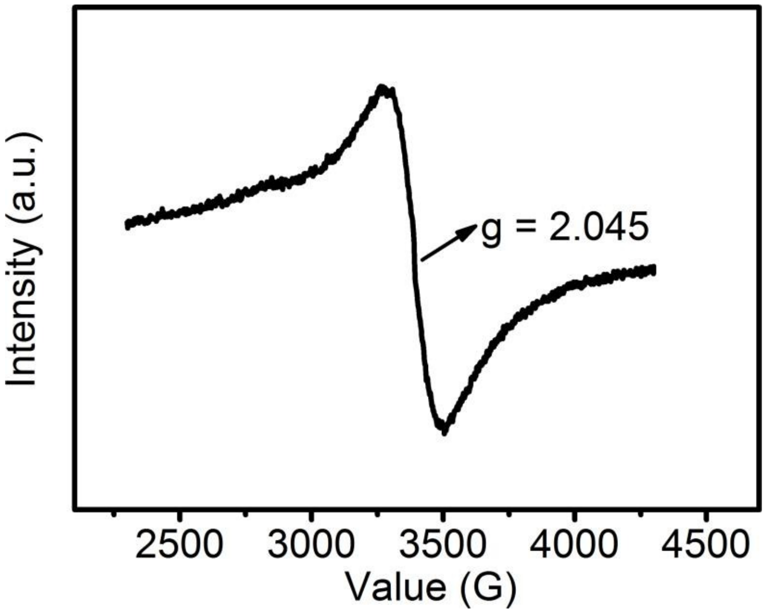

Figure S2. EPR spectrum of MnO.

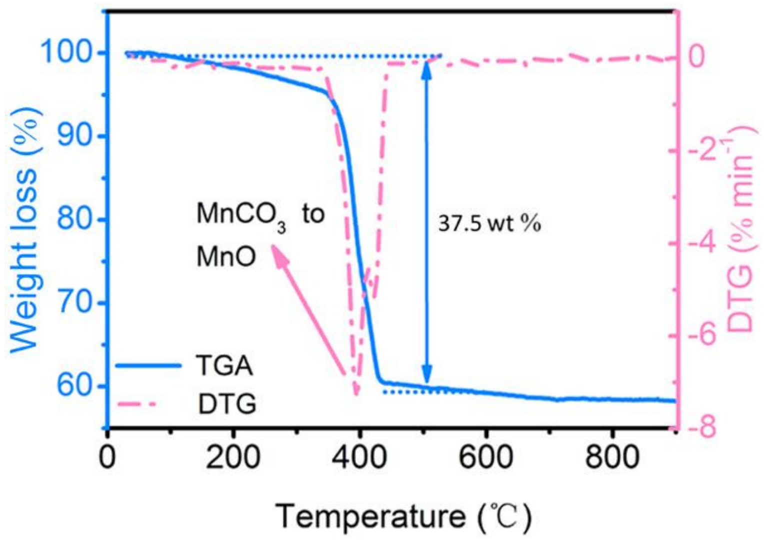

Figure S3. TGA and DTG curves of $MnCO_3$ under an Ar atmosphere.

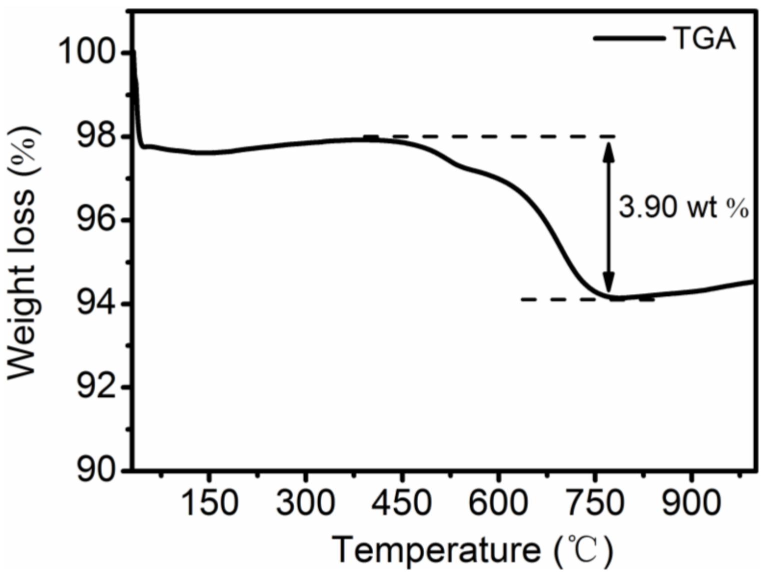

Figure S4. TGA curve of MnO under an Ar–air atmosphere.

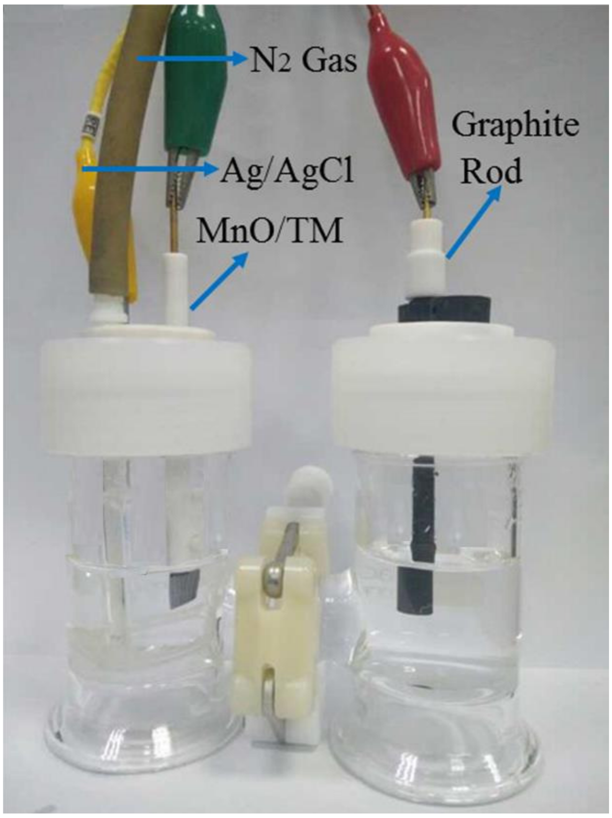

Figure S5. Optical photograph of the two-compartment electrochemical cell.

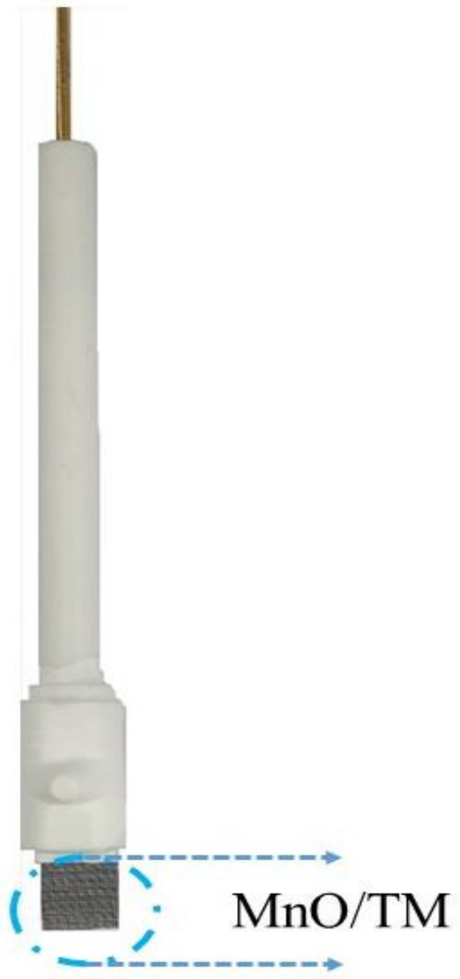

Figure S6. Optical photograph of the prepared cathode.

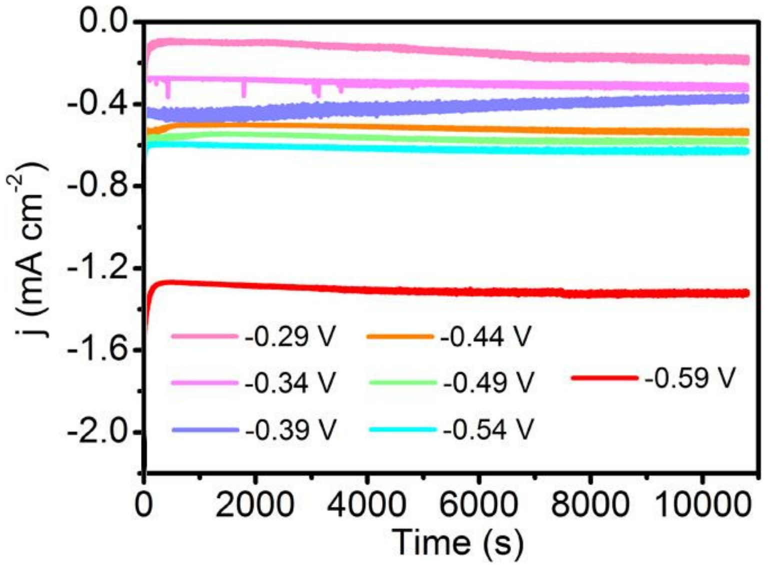

Figure S7. Time-dependent current density curves of MnO/TM for NRR at a series of potentials in 0.1 M Na_2SO_4.

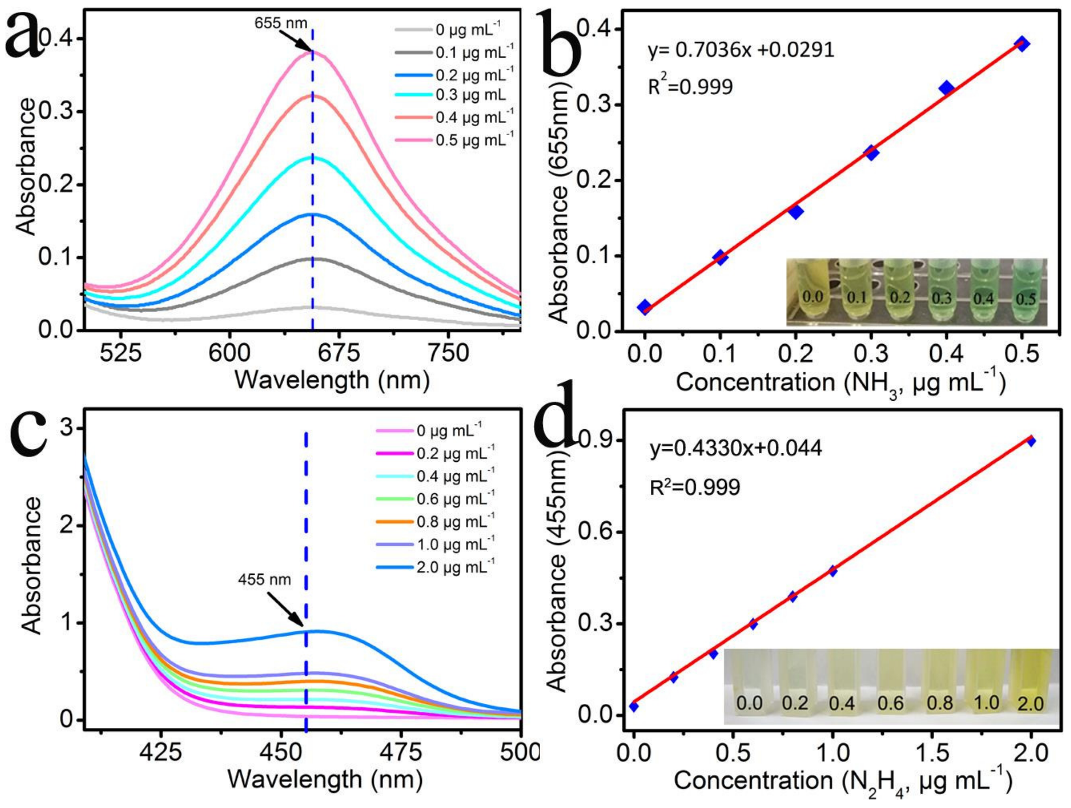

Figure S8. (a) UV-Vis absorption spectra of indophenol assays with NH_3 after incubated for 2 h at room temperature. (b) Calibration curve used for estimation of NH_3. (c) UV-Vis absorption spectra of various N_2H_4 concentrations after incubated for 15 min at room temperature. (d) Calibration curve used for calculation of N_2H_4·concentrations.

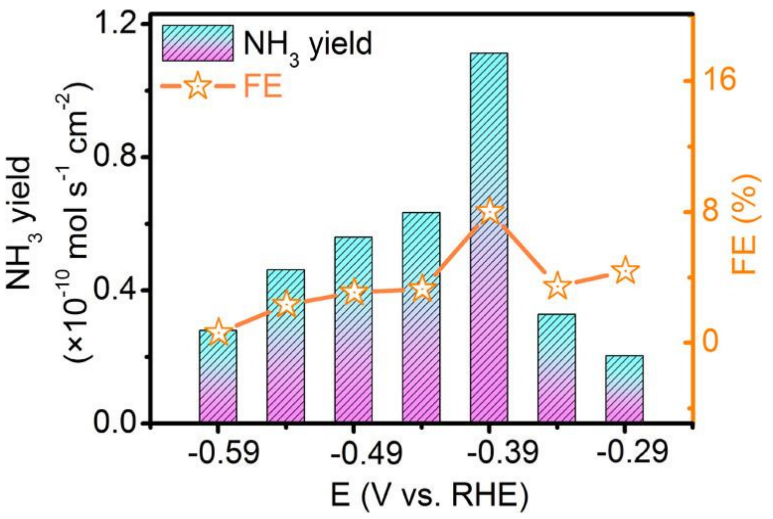

Figure S9. NH_3 yields and FEs of MnO/TM at a series of potentials for 3 h determined by ion chromatography.

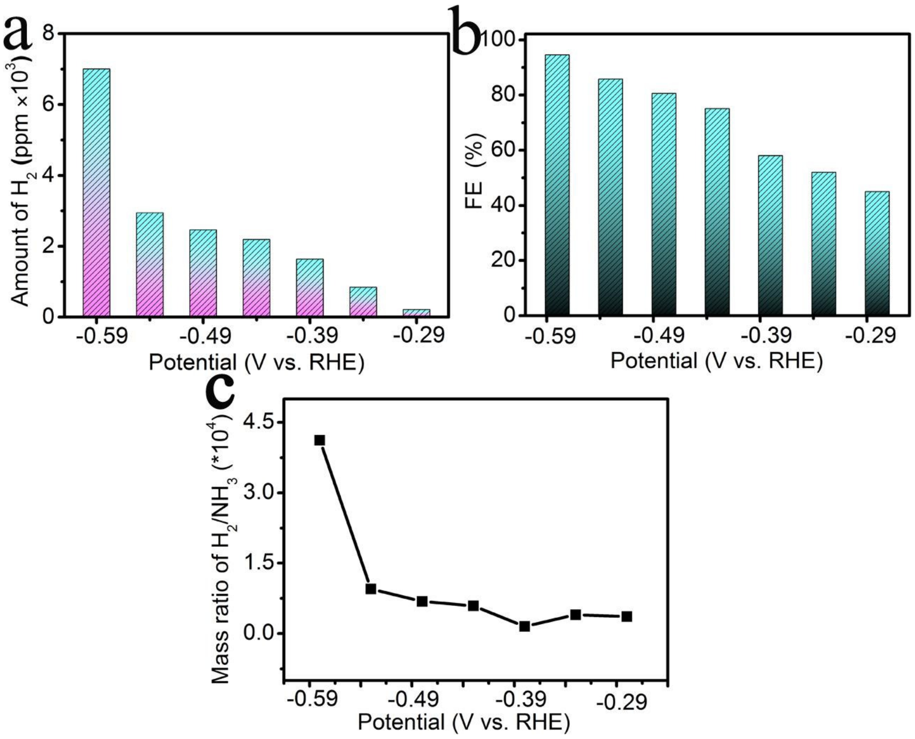

Figure S10. (a) Amount of evolved H_2 determined by gas chromatography from the headspace of the cell in N_2-saturated solutions at various potentials. (b) The calculated FEs of hydrogen formation at various potentials. (c) The mass ratio of H_2/NH_3 at various potentials.

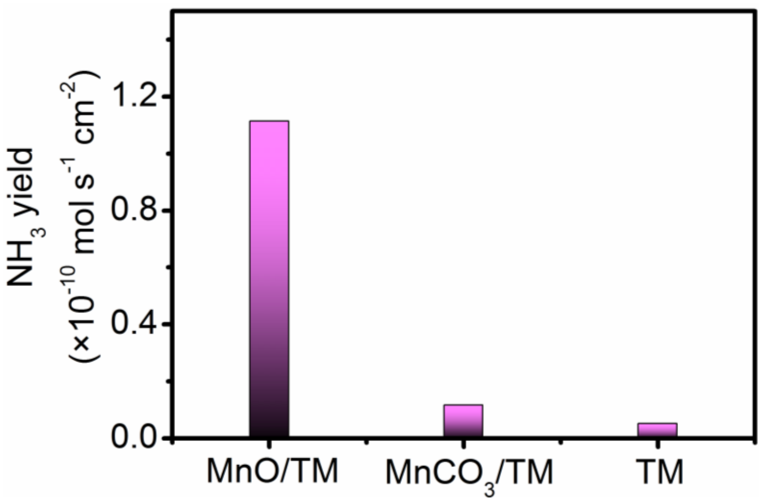

Figure S11. NH_3 yields for MnO/TM, $MnCO_3$/TM, and blank TM after 3 h electrolysis at –0.39 V.

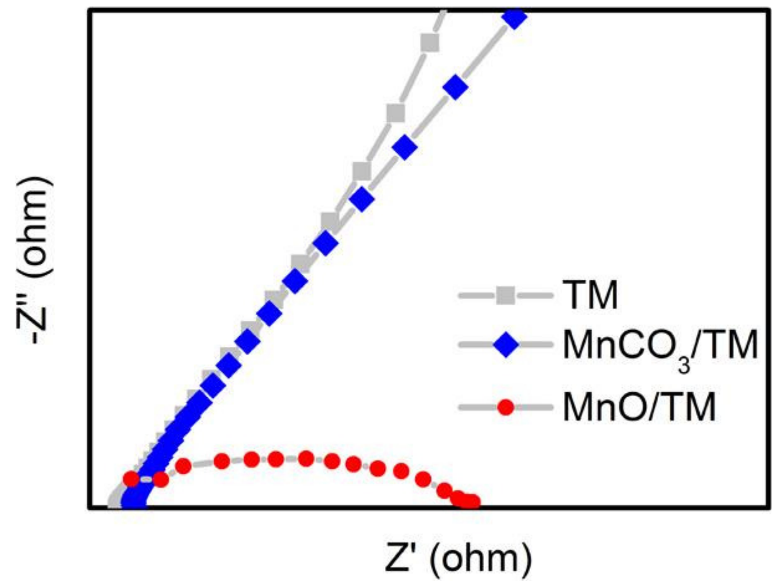

Figure S12. Nyquist plots of blank TM, $MnCO_3$/TM, and MnO/TM in 0.1 M Na_2SO_4 solution.

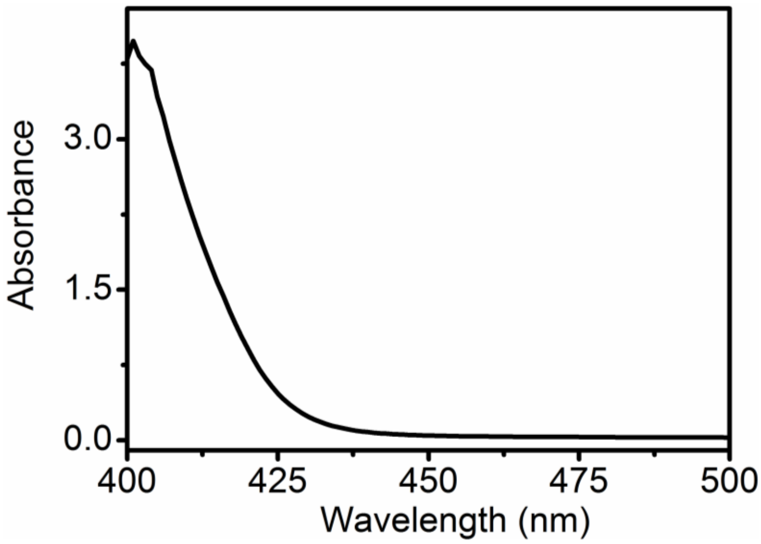

Figure S13. UV-Vis absorption spectrum of the electrolyte after electrolysis stained with para-(dimethylamino) benzaldehyde indicator after NRR electrolysis at –0.39 V.

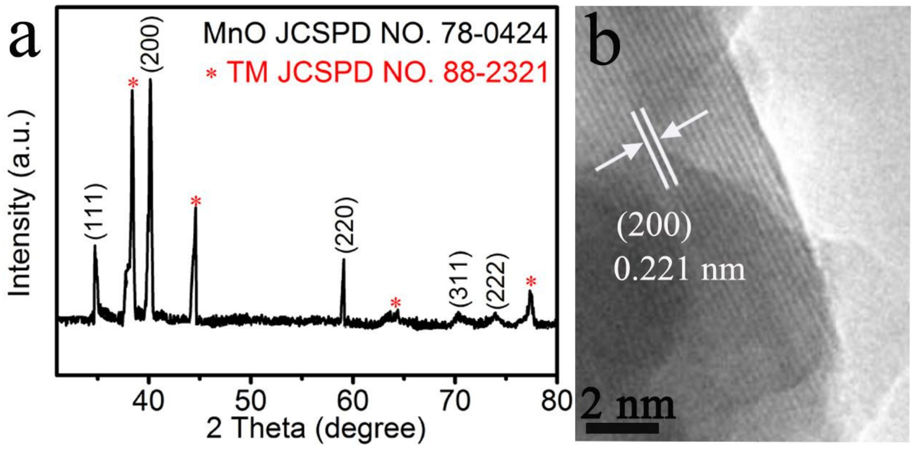

Figure S14. (a) XRD pattern of MnO/TM (b) HRTEM image of MnO after durability test.

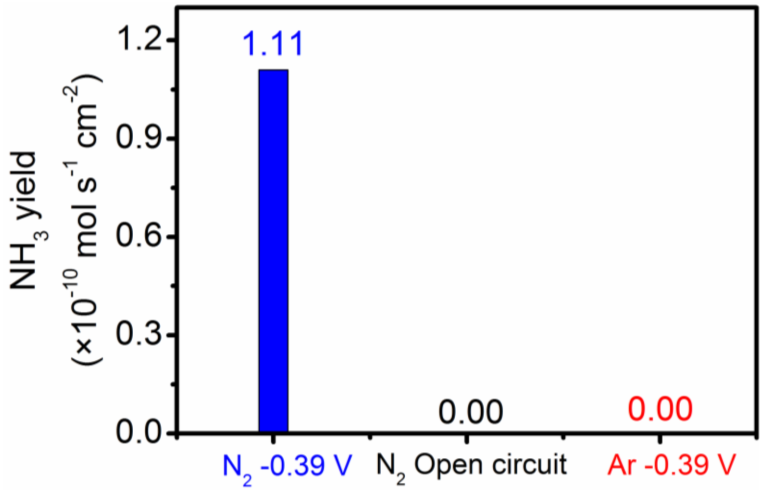

Figure S15. NH_3 yields for MnO/TM under N_2 gas at –0.39 V, under N_2 gas at an open-circuit, and under Ar gas at –0.39 V after 3 h electrolysis.

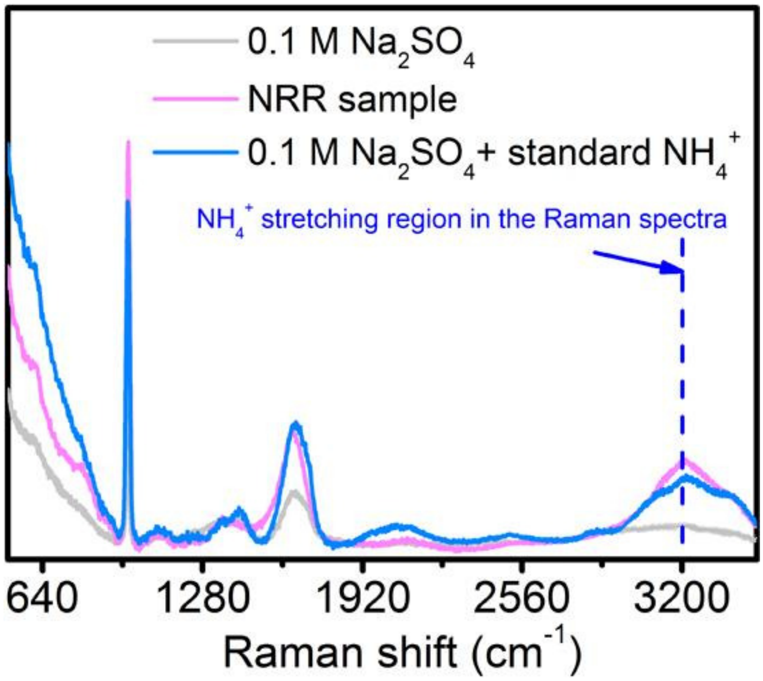

Figure S16. Raman spectra of 0.1 M Na_2SO_4, NRR sample, and 0.1 M $NaSO_4$ + standard NH_4^+.

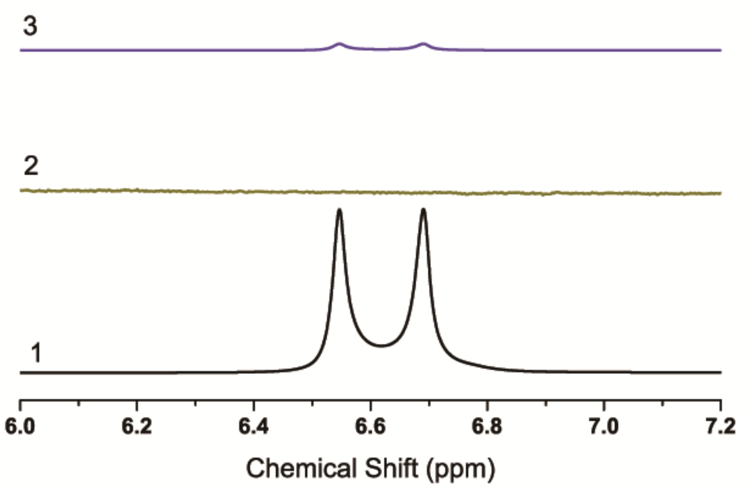

Figure S17. ^{15}N isotope labeling experiment. ^{1}H NMR spectra for $^{15}NH_4^+$ standard sample (curve 1) and electrolysis using Ar (curve 2) and $^{15}N_2$ (curve 3) as the feeding gas.

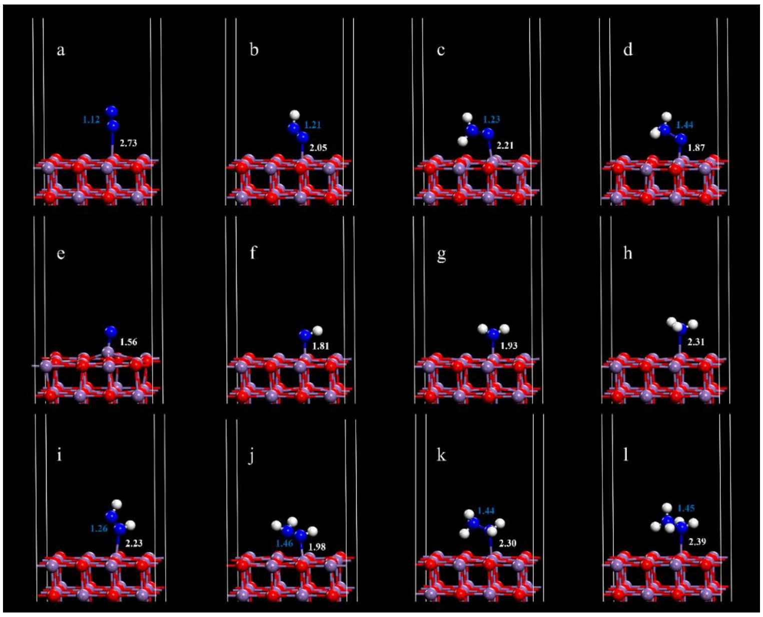

Figure S18. The optimized configurations of NRR on MnO (200) surface, (a–l) represents $*N_2$, *NNH, $*NNH_2$, $*NNH_3$, *N, *NH, $*NH_2$, $*NH_3$, *NHNH, $*NHNH_2$, $*NH_2NH_2$, and $*NH_2NH_3$, respectively.

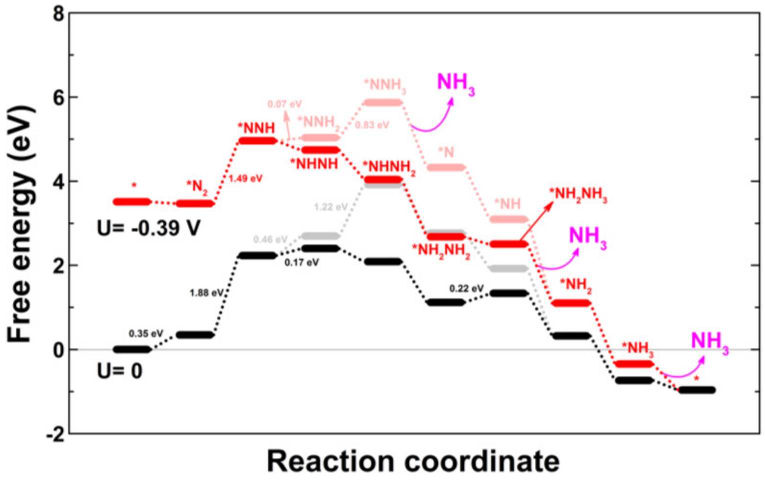

Figure S19. Free energy profile of NRR process on MnO (200) surface. The competitive processes are shown in light colors. Free energy change with an over potential of –0.39 V is marked in red.

Table S1. Data obtained from the ion chromatography for NH_3 concentrations after electrolysis for 3 h at a series of potentials.

**Sample**	**Potential (V vs. RHE)**	**Concentration (NH_3, ppm)**
1	–0.29	0.08
2	–0.34	0.21
3	–0.39	0.81
4	–0.44	0.37
5	–0.49	0.36
6	–0.54	0.31
7	–0.59	0.17

Table S2. Comparison of the electrocatalytic NRR performance of MnO/TM with other aqueous-based NRR electrocatalysts at ambient temperature.

Catalyst	Electrolyte	V_{NH3}	FE	Ref.
**MnO/TM**	**0.1 M Na_2SO_4**	**1.11×10^{-10} mol s^{-1} cm^{-2}**	**8.02%**	**This work**
		**7.92 μg h^{-1} $mg^{-1}_{cat.}$**		
		**6.72 μg h^{-1} cm^{-2}**		
Fe_2O_3-CNT	$KHCO_3$	3.58×10^{-12} mol s^{-1} cm^{-2}	0.15%	[1]
PEBCD/C	0.5 M Li_2SO_4	2.58×10^{-11} mol s^{-1} cm^{-2}	2.85%	[2]
MoS_2/CC	0.1 M Na_2SO_4	8.08×10^{-11} mol s^{-1} cm^{-2}	1.17%	[3]
Ru	2.0 M KOH	3.44×10^{-12} mol s^{-1} cm^{-2}	0.28%	[4]
Ru/Ti	0.5 M H_2SO_4	1.2×10^{-10} mol s^{-1} cm^{-2}	-	[5]
Rh/Ti		1.5×10^{-11} mol s^{-1} cm^{-2}		
Mo nanofilm	0.01 M H_2SO_4	3.09×10^{-11} mol s^{-1} cm^{-2}	0.72%	[6]
MoO_3	0.1 M HCl	4.80×10^{-10} mol s^{-1} cm^{-2}	1.9%	[7]
Mo_2N	0.1 M HCl	4.60×10^{-10} mol s^{-1} cm^{-2}	4.5%	[8]
TiO_2/Ti	0.1 M Na_2SO_4	9.16×10^{-11} mol $s^{-1}\cdot cm^{-2}$	2.5%	[9]
VN/TM	0.1 M HCl	8.40×10^{-11} mol s^{-1} cm^{-2}	2.25%	[10]
γ-Fe_2O_3	0.1 M KOH	0.212 μg h^{-1} $mg^{-1}_{cat.}$	1.9%	[11]
$Bi_4V_2O_{11}/CeO_2$	0.1 M HCl	23.21 μg h^{-1} $mg^{-1}_{cat.}$	10.16%	[12]
N-doped porous	0.05 M H_2SO_4	23.8 μg h^{-1} $mg^{-1}_{cat.}$	1.4%	[13]

carbon				
Au nanorods	0.1 M KOH	1.648 μg h^{-1} cm^{-2}	3.88%	[14]
B_4C	0.1 M HCl	26.57 μg h^{-1} $mg^{-1}_{cat.}$	15.95%	[15]
Nb_2O_5 nanofiber	0.1 M HCl	43.6 μg h^{-1} $mg^{-1}_{cat.}$	9.26 %	[16]
hollow Cr_2O_3 microspheres	0.1 M Na_2SO_4	25.3 μg h^{-1} $mg^{-1}_{cat.}$	6.78%	[17]
TiO_2-rGO	0.1 M Na_2SO_4	15.13 μg h^{-1} $mg^{-1}_{cat.}$	3.3%	[18]
Fe_2O_3 nanorods	0.1 M Na_2SO_4	15.9 μg h^{-1} $mg^{-1}_{cat.}$	0.94%	[19]
defect-rich MoS_2 nanoflower	0.1 M Na_2SO_4	29.28 μg h^{-1} $mg^{-1}_{cat.}$	8.34%	[20]

Table S3. Comparasion of the adsorption energy (E_{ads}), N-Mn bond length (d), free energy differenc (ΔE_f) of N_2 and NNH absorbed on the MnO (200) and $MnCO_3$ (104) surfaces.

	E_{ads} (eV)	d (Å)	ΔE_f (eV)
N_2/MnO (200)	–0.03	2.77	1.88
N_2/$MnCO_3$ (104)	–0.06	2.74	
NNH/MnO (200)	–0.57	2.04	1.98
N_2/$MnCO_3$ (104)	–0.63	2.02	

Table S4. The DFT calculated zero point energies $\mathbf{E_{ZPE}}$ and **T⊿S** of different adsorption species, where the * denotes the adsorption site on the MnO (200) surface. T is set as 27 °C.

**Adsorption Species**	$\mathbf{E_{ZPE}}$ **(eV)**	**T⊿S (eV)**
$\mathbf{*N_2}$	0.18	0.25
***NNH**	0.45	0.21
$\mathbf{*NNH_2}$	0.79	0.15
$\mathbf{*NNH_3}$	1.14	0.12
***NHNH**	0.81	0.13
$\mathbf{*NHNH_2}$	1.16	0.13
$\mathbf{*NH_2NH_2}$	1.26	0.17
$\mathbf{*NH_2NH_3}$	1.71	0.24
***N**	0.08	0.06
***NH**	0.29	0.07
$\mathbf{*NH_2}$	0.63	0.11
$\mathbf{*NH_3}$	0.95	0.08

References

[1] S. Chen, S. Perathoner, C. Ampelli, C. Mebrahtu, D. Su, G. Centi, Angew. Chem. Int. Ed. **2017**, 56, 2699–2703.

[2] G. Chen, X. Cao, S. Wu, X. Zeng, L. Ding, M. Zhu, H. Wang, J. Am. Chem. Soc. **2017**, 139, 9771–9774.

[3] L. Zhang, X. Ji, X. Ren, Y. Ma, X. Shi, Z. Tian, A. M. Abdullah, L. Chen, B. Tang, X. Sun. Adv. Mater. **2018**, 30, 1800191.

[4] V. Kordali, G. Kyriacou, C. Lambrou, Chem. Commun. **2000**, 17, 1673–1674.

[5] K. Kugler, M. Luhn, J. A. Schramm, K. Rahimi, M. Wessling, Phys. Chem. Chem. Phys. **2015**, 17, 3768–3782.

[6] D. Yang, T. Chen, Z. Wang, J. Mater. Chem. A **2017**, 5, 18967–18971.

[7] J. Han, X. Ji, X. Ren, G. Cui, L. Li, F. Xie, H. Wang, B. Li, X. Sun, J. Mater. Chem. A **2018**, 6, 12974–12977

[8] X. Ren, G. Cui, L. Chen, F. Xie, Q. Wei, Z. Tian, X. Sun, Chem. Commun. **2018**, 54, 8474–8477

[9] R. Zhang, X. Ren, X. Shi, F. Xie, B. Zheng, X. Guo, X. Sun, ACS Appl. Mater. Interfaces **2018**, 10, 28251–28255.

[10] R. Zhang, Y. Zhang, X. Ren, G. Cui, A. M. Asiri, B. Zheng, X. Sun, ACS Sustainable Chem. Eng. **2018**, 6, 9545–9549.

[11] J. Kong, A. Lim, C. Yoon, J. H. Jang, H. C. Ham, J. Han, H. S. Park, ACS Sustainable Chem. Eng. **2017**, 5, 10986–10995.

[12] C. Lv, C. Yan, G. Chen, Y. Ding, J. Sun, Y. Zhou, G. Yu, Angew. Chem. Int. Ed. **2018**, 57, 6073−6076.

[13] Y. Liu, Y. Su, X. Quan, X. Fan, S. Chen, H. Yu, H. Zhao, Y. Zhang, J. Zhao, ACS Catal. **2018**, 8, 1186−1191.

[14] D. Bao, Q. Zhang, F. Meng, H. Zhong, M. Shi, Y. Zhang, J. Yan, Q. Jiang, X. Zhang, Adv. Mater. **2017**, 29, 1604799.

[15] W. Qiu, X. Xie, J. Qiu, W. Fang, R. Liang, X. Ren, X. Ji, G. Cui, A. M. Asiri, G. Cui, B. Tang, X. Sun, Nat. Commun. **2018**, 9, 3485.

[16] J. Han, Z. Liu, Y. Ma, G. Cui, F. Xie, F. Wang, Y. Wu, S. Gao, Y. Xu, X. Sun, Nano Energy **2018**, 52, 264–270.

[17] Y. Zhang, W. Qiu, Y. Ma, Y. Luo, Z. Tian, G. Cui, F. Xie, L. Chen, T. Li, X. Sun, ACS Catal. **2018**, 8, 8540–8544.

[18] X. Zhang, Q. Liu, X. Shi, A. M. Asiri, Y. Luo, T. Li, X. Sun, J. Mater. Chem. A **2018**, 6, 17303–17306.

[19] X. Xiang, Z. Wang, X. Shi, M. Fan, X. Sun, ChemCatChem **2018**, DOI: 10.1002/cctc.201801208.

[20] X. Li, T. Li, Y. Ma, Q. Wei, W. Qiu, H. Guo, X. Shi, P. Zhang, A. M. Asiri, L. Chen, B. Tang, X. Sun, Adv. Energy Mater. **2018**, 8, 201801357.
